# The Crucial Role of Advanced Image-Guided Brachytherapy for Locally Advanced Cervical Cancer in the Era of Systemic Treatment Intensification

**DOI:** 10.3390/cancers17111809

**Published:** 2025-05-28

**Authors:** Dina Najjari-Jamal, Marc Juarez, Sofia Cordoba, Francisco Celada, Milica Stefanovic, Cristina Gutierrez

**Affiliations:** 1 Department of Radiation Oncology, Catalan Institute of Oncology, University of Barcelona, Hospitalet de Llobregat, 08908 Barcelona, Spain; mjuarez@iconcologia.net (M.J.); milicastefanovic@iconcologia.net (M.S.); or cristina.gutierrez@hmar.cat (C.G.); 2 Department of Radiation Oncology, Puerta de Hierro University Hospital, 28222 Majadahonda, Spain; sofia.cordoba@salud.madrid.org; 3 Department of Radiation Oncology, Hospital Universitario y Politécnico La Fe, 46026 Valencia, Spain; celada_fra@gva.es; 4 Department of Radiation Oncology, Hospital del Mar, 08003 Barcelona, Spain

**Keywords:** cervical cancer, LDR, HDR, PDR, brachytherapy, immunotherapy

## Abstract

Background/Objectives: Recent advancements in the treatment of LACC have focused on improving outcomes through systemic treatment intensification. Therefore, this review aims to analyze the brachytherapy (BT) techniques employed in recent studies that are likely to change upcoming clinical guidelines, and to discuss the evolving role of IGABT in optimizing patient outcomes. Methods: This review focuses on BT practices reported across main phase III trials—OUTBACK, INTERLACE, CALLA, and KEYNOTE A18—compared with the EMBRACE I study. Analyzed parameters include BT modality, dose prescription techniques, imaging guidance, and overall treatment time (OTT). Results: In EMBRACE I, MRI-based IGABT was mandatory, with 43% of patients receiving an intracavitary/interstitial (IC/IS) applicator; cumulative EQD2 D90 HR-CTV was 90 Gy with a median OTT of 46 days. The OUTBACK trial relied predominantly on point A-based BT, with limited use of volume-based BT (28%). The INTERLACE trial reported mixed BT approaches: 70% point A-based, 30% volume-based, and 20% 2D BT. A median cervical dose of 79.4 Gy was reported. CALLA maintained strong protocol adherence, with 60% volume-based BT and a median tumor EQD2 dose of 83 Gy, although lower in the Japan cohort. In the KEYNOTE A 18 cohort, volume-based BT was performed in 88% of patients, with a median D90 HR-CTV dose of 87 Gy; IC/IS applicators were used in 23% of cases. Conclusions: Across these major studies, the following consistent pattern emerges: the quality and technique of BT impact survival outcomes and toxicity profile in LACC. MRI-based IGABT—with the use of IC/IS applicators when needed—is essential.

## Simple Summary

In the last two decades, advancements in the treatment of locally advanced cervical cancer (LACC) have been limited. Technological progress has enhanced radiotherapy techniques, particularly through image-guided adaptive brachytherapy (IGABT), significantly enhancing local control rates. Despite these improvements in standard LACC treatment, systemic therapy has predominantly relied on weekly cisplatin. Recent attempts to intensify chemotherapy regimens have yielded positive outcomes. However, these results should be interpreted with caution, as the planned brachytherapy treatment did not meet high-quality standards. In this article a comprehensive analysis of the patient and treatment characteristics was conducted, with particular emphasis on the critical role of maintaining high-quality IGABT within the standard treatment for LACC.

## 1. Introduction

In the last 25 years, the treatment of locally advanced cervical cancer (LACC) has significantly improved due to technological advances in radiotherapy (RT) [1,2,3]. This improvement is reflected in the results of the prospective multicenter EMBRACE I study (IntErnational Study on MRI-Based BRAChytherapy in Cervical Cancer), which confirmed the value of image-guided adaptive brachytherapy (IGABT) in LACC [4]. In patients treated with IGABT, the 5-year overall survival (OS) rate was 74%, with a local control (LC) rate > 90% in all stages of the disease [3,4,5,6]. The OS rate was significantly better than the historical data, with less locoregional and distant recurrence [7]. Crucially, these excellent results were achieved without any increase in treatment-related toxicity or deterioration in quality of life, and IGABT now forms part of the standard treatment of LACC [8,9,10,11,12].

Moreover, in contrast with the improvement in RT techniques, systemic therapy has remained largely unchanged since the publication of five large randomized trials in the late 1990s [13,14,15,16,17]. A meta-analysis of those trials showed that the addition of cisplatin-based chemotherapy (ChT) improved OS and progression-free survival (PFS) rates by 6% and 8%, respectively. Nevertheless, the addition of concomitant ChT added only a modest 3% improvement of disease-free survival (DFS) for patients with stage IIIB cancer [18]. A randomized study conducted in India further demonstrated that concurrent cisplatin in combination with RT and volume-based brachytherapy (BT) notably enhanced outcomes for stage IIIB, raising the 5 y OS rate from 46% to 64% [19]. In the EMBRACE-I cohort, the implementation of IGABT contributed to a 14–17% increase in both LC and pelvic control among patients with FIGO 2014 stage IIIB-IVA disease [4].

In this line, Shmidt et al. demonstrated the dose–effect relationships in LACC based on data from the 1318 EMBRACE-I patients. An increase in the total D90 high-risk clinical target volume (HR-CTV) dose was associated with improved local control, emphasizing the importance of adequate dose escalation, particularly for large tumor volumes and non-squamous histologies [6].

Few data have consistently demonstrated that tumor volume and overall treatment time (OTT) are independent prognostic factors influencing LC [9,20,21]. Despite the implementation of advanced IGABT and concurrent ChT, prolonged OTT remains a significant detriment to local outcomes. Excessive OTT (>55 days) and larger HR-CTV volumes are independent adverse prognostic factors, as confirmed in a multi-institutional study involving 488 patients treated with RTCHT and MRI-based IGABT. Moreover, each additional week of treatment delay or a 10 cc increase in HR-CTV volume necessitated a dose escalation of 5 Gy to maintain equivalent rates of local control [9].

Despite advancements in RT techniques, particularly in BT, the standard treatment for LACC is not globally widespread. Limited resources and accessibility, especially in low- and middle-income countries, hinder the standardization of IGABT in these regions, contributing to increased incidence and mortality from LACC [22]. Moreover, IGABT for cervical cancer is a complex treatment requiring many new skills with varying learning needs. In recent years, new educational strategies have been developed to address some of these limitations [23]. In this context, recent phase III randomized trials have examined the impact of treatment intensification, yielding mixed results [24,25,26,27,28]. These large multicenter and international studies, involving a wide range of recruiting sites, have faced challenges in consistently achieving high-quality RT standards, particularly given the steep learning curve associated with the implementation of IGABT, even within experienced centers.

Given the importance of IGABT in the management of LACC, this review aims to analyze the BT techniques employed in recent studies that are likely to change upcoming clinical guidelines, and to discuss the evolving role of IGABT in optimizing patient outcomes.

## 2. Materials and Methods

A comprehensive review was conducted to assess randomized controlled trials (RCTs) investigating treatment strategies for patients with LACC. Eligible studies included phase III randomized trials that evaluated radiochemotherapy (RTCHT) with BT, combined with additional systemic therapies such as adjuvant, neoadjuvant, or concurrent chemotherapy, targeted agents, or immunotherapy. Trials that compared these strategies with the standard-of-care treatment—defined as external beam radiotherapy (EBRT) with concurrent platinum-based CHT and BT—were included.

Only peer-reviewed full-text articles published in English were considered. Abstracts from conference proceedings, non-randomized studies, retrospective analyses, meta-analyses, narrative reviews, editorials, letters to the editor, and case reports were excluded. In addition, studies focusing exclusively on recurrent, persistent, or metastatic disease, or on early-stage cervical cancer managed primarily with surgery, were not eligible for inclusion.

Given that the IGABT concept was introduced on the recommendations of the Gynecological (GYN) Groupe Europeen De Curietherapie-European Society for Therapeutic Radiology and Oncology (GEC-ESTRO) in 2005 and became more standardized in 2016, with the publication of the RetroEMBRACE results, trials started before the year 2005 were excluded.

## 3. Results

After the search strategy and eligibility assessment, a total of four articles met the inclusion criteria (Table 1). We sought to include EMBRACE studies for methodology comparison purposes.

### 3.1. EMBRACE I

The EMBRACE-I study is a prospective multi-institutional longitudinal study on MRI-based IGABT in addition to concurrent RTCHT in LACC. Eligibility criteria were female patients affected by squamous, adenocarcinoma, or adenosquamous cell carcinoma, FIGO (2009) stage IB-IVA and IVB, restricted to paraaortic lymph node metastases. Up to 76.7% of the patients were staged IIB or more, and 52% were node-positive at diagnosis. In total, 1341 patients were available for analysis. Concomitant chemotherapy was weekly cisplatin 40 mg/m^2^, 5–6 cycles (91.6%). Permitted EBRT techniques were 3D or intensity modulated RT (IMRT)/volumetric arc therapy (VMAT), used in 58.8% and 41% of cases, respectively. Magnetic resonance imaging (MRI)-based IGABT was mandatory for at least the first implant application. The BT technique was intracavitary (IC) (56.6%) or a combination of intracavitary plus interstitial (IC/IS) component (43%). In 57% of cases, the high dose rate (HDR) was used, and, in 41.9%, pulsed dose rate (PDR) was the preferred option. The target volume and dose reporting of HR-CTV and intermediate risk clinical target volume (IR-CTV) and the organs at risk (OAR) followed the gynecological (GYN) GEC-ESTRO recommendations. The cumulative equivalent dose in 2 Gy fractions (EQD2) in 90% of the volume (D90) of the HR-CTV was 90 Gy (85–94), the median overall treatment time (OTT) was 46 days (range 42–50). With a median follow-up of 51 months (ICR 20–64), 5-year local, pelvic, and nodal control were 92%, 87%, and 87%, respectively. Global 5-year OS was 74%, and 5-year disease-free survival was 68%. Grade ≥ 3 late toxicity was 14.6%, with 3.2% of fistulae [4].

### 3.2. OUTBACK Trial

From April 2011 to June 2017, the OUTBACK trail randomized 926 patients with LACC to receive either standard RTCHT or RTCHT followed, within 4 months, by adjuvant chemotherapy consisting of up to four cycles of carboplatin AUC5 and paclitaxel (155 mg/m^2^). The primary endpoint was 5 y OS. The study included patients with squamous cell carcinoma, adenosquamous carcinoma, or adenocarcinoma, staged as IB1 node-positive, IB2, II, IIIB, and IVA FIGO 2008. Of the total cohort, 76% were staged IB1 and node-positive IIB. Key exclusion criteria were stage IIIA disease and tumor size at diagnosis requiring interstitial BT. Approximately 20% of the patients allocated to adjuvant chemotherapy did not receive any cycle, primarily due to patient preference. Regarding RT, cobalt 60 units were permitted, and 92% were treated with the four field box (3D) technique. EBRT was completed without interruption in 92% of patients, and BT was administered in 95%, predominantly via intracavitary tandem applicators. HDR BT was used in >90% of cases across both arms. Although the recommended cumulative EQD2 dose ranged from 80 to 86.4 Gy, no data on median dose delivered were reported. Parametrial boosts were given to 36% of the patients. Despite the feasibility of volume-based prescriptions, 64% of patients received BT prescribed to point A, while 28% were treated with volume-based BT. The OTT exceeded 56 days in approximately 35% of cases. After a median follow-up of 60 months (IQR 45–65), 5 y OS was comparable between groups (72 vs. 71%; *p* = 0.81%). However, the incidence of serious adverse events was higher in the adjuvant chemotherapy group (30% vs. 22%) [25].

### 3.3. INTERLACE Trial

This academic trial aimed to compare the addition of induction chemotherapy (IChT) with carboplatin/paclitaxel 6-weekly cycles to the standard RTCHT and BT treatment in LACC. Over 10 years, 500 patients were randomized to either the IChT arm or the placebo arm. The final results of the INTERLACE trial were recently published. The 5-year OS rate was 80% in the experimental arm versus 72% in the control arm (hazard ratio: 0.60 [95% CI: 0.40–0.91; *p* = 0.015]). At diagnosis, 86% of patients were stage IB2-IIB (FIGO 2014 criteria) and only 43% of the cohort had lymph node involvement at diagnosis. Adherence to concomitant cisplatin was lower in the experimental arm than in controls (68% vs. 79%), and only 85% of these patients completed all four cycles. The incidence of severe (G3–4) hematological toxicity was also substantially higher in the experimental arm (25% vs. 13%). Overall, 96% and 97% in the control arm completed EBRT and BT, respectively. Approximately 60% of the cohort received EBRT planned with 3D techniques, while the remainder were treated with IMRT. All centers underwent RT quality assurance assessments before and during participation. Volume-based BT was planned for 30% of patients, although it was not specified whether imaging was performed with MRI or CT. The remaining 70% of patients in both groups were treated with point A-based BT, with 49% using 3D imaging and 20% using 2D techniques. The use of IC/IS applicators was permitted, although no information on their usage rates within the cohort was described. The mean dose to the cervical tumor across the full cohort was 79.4 Gy, with 13% of patients in both groups receiving less than 70.2 Gy. By contrast, the mean doses were substantially higher in the volume-based BT subgroup (87 Gy). Median OTT for RTCHT + BT was 45 days. The interval between the completion of IChT and the start of RTCHT was ≤7 days in 78% of patients, and ≤14 days in 93% [26].

### 3.4. CALLA Trial

In the CALLA trial, 770 patients staged Ib2-IIB node-positive and IIIA-IVA, regardless of node status (FIGO 2009), were randomized to receive either placebo or durvalumab concurrently with RTCHT and as maintenance treatment. This RCT concluded that durvalumab combined with the standard treatment did not significantly improve PFS compared with placebo, with a PFS at 1 year of 76% in the durvalumab group vs. 73.3% in the placebo group (HR: 0.84; [95% CI: 0.65–1.08; *p* = 0.17]). Regarding treatment characteristics, BT practices remained consistent and rigorous across treatment arms. Notably, a Japan-specific RT protocol was developed due to differences in local standard-of-care target doses and guidelines, which tend to be lower compared with other countries. Most patients received HDR treatment (88.1%), while a smaller proportion received low-dose rate (LDR) BT (7.6%). BT techniques varied, with volume-based BT used in approximately 60% of the patients globally. The remaining patients received standard point-directed methods, employing either 2D planar imaging (25%) or volumetric planning for organ-at-risk delineation (8%). In Japan, volume-directed BT was even more prevalent, utilized in about 70% of patients. Protocol adherence was high, with 94.3% and 93.5% in the durvalumab and placebo arms, respectively, completing the BT dose per protocol. OTT was ≤59 days in 72% of patients and ranged from >60 to ≤70 days in 13.5% of patients. The global population achieved a median EQD2 of 83 Gy (IQR 83–87 Gy); however, in Japan, the median EQD2 dose to the tumor was 70 Gy (IQR 65–73 Gy), reflecting regional protocol adjustments. Additionally, 86% of the overall study population received parametrial boost [27].

### 3.5. KEYNOTE A18

The ENGOT-cx11/GOG-3047/KEYNOTE-A18 trial was a phase III randomized double-blind placebo-controlled study that evaluated the addition of an anti-PD-1 (pembrolizumab) to standard RTCHT + BT, regardless of PDL-1 status. Between 2020 and 2022, a total of 1060 patients were enrolled. Inclusion criteria were diagnosis of squamous, adenosquamous, or adenocarcinoma of the cervix, and considered high risk, defined as FIGO 2014 stage IB2-IIB node-positive (defined as a short-axis diameter of ≥15 mm), or stage III-IVA regardless of nodal status. Approximately 56% of patients in both arms were staged as III-IVA, and 86% had positive lymph nodes (measured as 15 mm in the short axis). In the experimental arm, patients received 5 cycles of pembrolizumab every 3 weeks concurrently with RTCHT (weekly cisplatin 40 mg/m^2^), followed by 15 maintenance cycles of pembrolizumab every 6 weeks. Patients were stratified at randomization by a planned EBRT technique (IMR/VMAT vs. non-VMAT) and staged at screening (IB2/IIB node-positive vs. III-IVA). Stratification by total EQD2 dose (<70 Gy or > or equal 70 Gy) was also made to consider Japanese protocols and guidelines. Pelvic EBRT was delivered using the IMRT/VMAT technique in 88% of cases, while conformal 3D RT was employed in 11%. Regarding BT, HDR was the preferred modality. Volume-based BT with HR-CTV definition and dose prescription according to ICRU report 89 was performed in 88% of patients. The remaining 9% received a point A-based 3D technique. Notably, a combined IC/IS BT applicator was used in 23% of patients across both arms. The median total EQD2 D90 to the HR-CTV was 87 Gy (IQR 83–92). OTT was 52 days (IQR 49–57), though only 35% of patients completed treatment within the recommended 50 days.

With a median follow-up of 29.9 months (IQR 23.3–34.3), data from the second interim analysis showed a significant improvement in PFS (69.3% vs. 56.9%) and OS (82.6% vs. 74.8%), and a hazard ratio for death of 0.67 (95% CI: 0.50–0,90; *p* = 0.0040). Grade ≥ 3 acute adverse events occurred in 78% of the pembrolizumab/RTCHT group and 70% of the placebo/RTCHT group. The forest plot for OS showed that patients treated with IMRT/VMAT benefited from pembrolizumab [HR 0.67 (CI95% 0.47–0.94)], along with those with total doses > 70 Gy [HR 0.68, CI95% 0.50–0.92)]. The forest plot for PFS also showed that patients treated with IMRT/VMAT and doses > 70 Gy had better outcomes with pembrolizumab, with HR 0.66 (CI95% 0.53–0.82) and 0.68, (CI95% 0.55–0.85), respectively [24,30].

## 4. Discussion

BT remains a main component in the curative management of LACC, as robustly demonstrated in several trials [4,19,31]. Despite advances in systemic treatment and EBRT techniques, optimized BT remains irreplaceable for achieving LC and improving survival outcomes [6,32].

In the 2000s, the GEC-ESTRO working group published the recommendations in target contouring, implant technique, and treatment planning with MRI-based IGABT [1,2,33], and these recommendations have been undertaken in the ICRU report 89.

**Figure 1:**
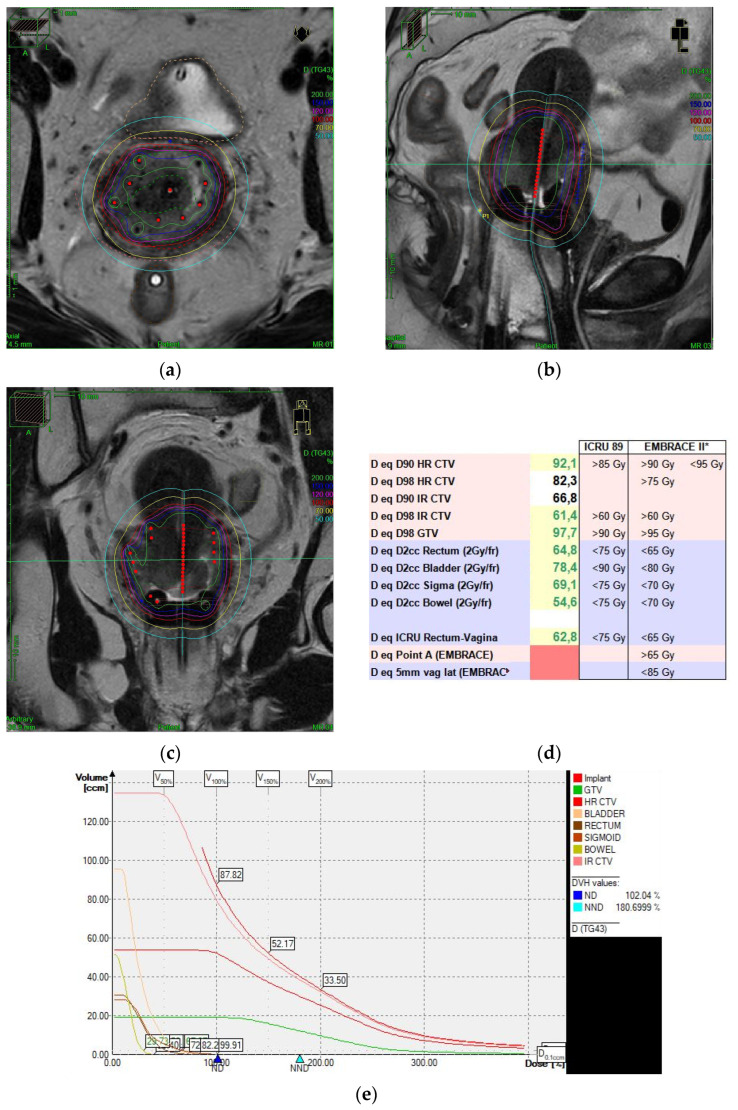
Image-guided adaptive brachytherapy (IGABT) for a patient with HPV-related squamous cell carcinoma of the cervix, T2bN2M0 FIGO 2018 stage IIIC2. Concomitant radiochemotherapy was delivered with 45 Gy in 25 fractions of 1.8 Gy, followed by high dose rate (HDR) MRI-guided IGABT in 4 fractions of 7 Gy/fraction, administered in two separate implants. A combined intracavitary/interstitial (IC/IS) technique was performed, using 10 round interstitial needles (8 parallel and 2 oblique). Panels (**a**–**c**) show axial, sagittal, and coronal views of the implant dosimetry using Oncentra^®^ Brachy version 4.6.3 (Elekta AB, Stockholm, Sweden), respectively. The images show full coverage of the large residual tumor and cervix with IC/IS implant, while sparing organs at risk (bladder, rectum, and sigmoid). Panel (**d**) displays the cumulative EQD2 doses, with D90 to the high-risk clinical target volume (HR-CTV) reaching 92.1 Gy. Panel (**e**) shows the dose–volume histogram (DVH) for the brachytherapy plan. The image was edited by the author and is reproduced with permission from the Radiation Oncology Department at ICO Hospitalet de Llobregat. * Planning aim and constraint recommendations based on the EMBRACE II study.

The EMBRACE -I study set a new benchmark by establishing MRI-IGABT as a critical component of LACC management. With a median OTT of 46 days and a cumulative EQD2 D90 to the HR-CTV of 90 Gy, 5 y LC was 90% (95% CI: 90–93), with 5 y OS of 74% (95 CI: 72–77) across all stages. Notably, the frequent need for an IC/IS component (43%) underscores the necessity for individualized volume-based dose escalation to achieve optimal outcomes while sparing OAR, introducing the concept of advanced high-quality IGABT [4] (Figure 1).

Historically, the phase III chemotherapy trials that have been performed to improve outcomes in cervical cancer have reported negative results, with treatment intensification associated with increased toxicity [34,35]. In addition, data regarding BT procedures, techniques, and dose reports from these trials are scarce. Therefore, this review aims to analyze the BT techniques and quality across the main RCTs in LACC in the IGABT era.

In the phase III OUTBACK trial, which assessed the addition of adjuvant ChT after standard RTCHT and BT, did not show a survival benefit (OS at 5 years 72% vs. 71%), but rather an increased 8% in G3 late events compared with the control arm [25]. Noticeably, the control arm had 22% of G3 or more late events, which contrasts with the results of EMBRACE-I, where less than 15% of patients experienced a serious late toxicity [12]. Although BT was delivered to 95% of patients, the predominant use of point A-based prescription (64%) and the absence of interstitial techniques—which likely impacted the total dose to the HRCTV (median doses not reported)—probably contributed to suboptimal LC and resulted in an increased incidence of late toxicity.

The INTERLACE trial showed a 5-year OS rate of 80% in the experimental arm versus 72% in the control arm (hazard ratio: 0.60 [95% CI: 0.40–0.91; *p* = 0.015]). However, it is important to interpret these findings in the context of the important drawbacks of that trial, which may invalidate the applicability of these findings to current clinical practice. At diagnosis, 86% of patients were stage IB2-IIB (FIGO 2014 criteria), and only 43% of the cohort had lymph node involvement at diagnosis [26]. In other words, most of these patients had a good prognosis, as demonstrated by Lindegaard et al., who compared the INTERLACE cohort with a subgroup of patients from the EMBRACE I trial with a comparable clinical profile (n = 1141) (denominated the “INTERLACE-like” subgroup). The five-year OS rate in the “INTERLACE-like” subgroup from the EMBRACE I was 78%, in line with the results of the INTERLACE study [36]. This analysis suggests that the INTERLACE findings may not apply to patients with a higher risk of recurrence. Moreover, patients with positive para-aortic lymph nodes were excluded from the INTERLACE trial, which raises doubts about the study’s representativeness in high-risk populations, thus further compromising the relevance of those findings for high-risk patients. Nevertheless, a large proportion of patients (70%) did not receive volume-based BT, and a notable fraction still managed with 2D techniques. Moreover, the impact of IGABT in the INTERLACE trial was evident in the mean dose to the cervical tumor, which was 79.4 Gy at point A in the full cohort (i.e., patients treated with and without image-guided BT). By contrast, the mean doses were substantially higher in the IGABT subgroup (87 Gy), which underscores the important differences between groups in terms of dose and coverage. Clearly, the relatively low dose at point A is insufficient to achieve full coverage, especially in bulky tumors.

Lindegaard et al. found that the survival curves of the INTERLACE-like subgroup (EMBRACE I) and the INTERLACE cohort overlapped, leading them to conclude that neoadjuvant chemotherapy in the INTERLACE trial merely served to compensate for the suboptimal RT [36]. While IChT may be a valid strategy in centers that lack access to standard treatment modalities, the application of suboptimal local treatment is not acceptable in routine practice. Rather, all centers should strive to offer the highest quality local treatment in order to ensure the best possible results [37].

The combination of standard RTCHT + BT and immune checkpoint inhibitors (ICIs) has been tested in LACC due to its relationship with HPV infection and its immunosuppressive environment [38,39]. The CALLA trial, published in 2023, explored the combination of durvalumab with concurrent standard RTCHT and maintenance up to 2 years. The promising study was found to be negative in its primary endpoint, with a similar PFS in both groups. Consideration must be taken of the multiple factors influencing the results. Approximately 28% and 24% of patients in the experimental and control arms were node-negative, who, as demonstrated in the forest plot, did not benefit from durvalumab [27]. Despite the findings from the EMBRACE study, where a total EQD2 dose of at least 85 Gy was related to higher LC, the CALLA trial reported median doses of 83 Gy and 70 Gy for non-Japanese and Japanese populations, respectively [6]. These lower doses may have influenced the study outcomes, particularly in the control arm. Interestingly, no dose stratification was made in the forest plot analysis, limiting the ability to assess dose–response relationships within the study population. Additionally, approximately 40% of patients were treated using conventional BT techniques, and it can be extrapolated that 86% of the cohort received a parametrial boost, underscoring the limited use of interstitial BT within the trial.

In contrast, the phase III ENGOT-cx11/GOG3047/KEYNOTE-A18 trial showed a significant improvement in PFS (69.3% vs. 56.9%) and OS (82.6% vs. 74.8%) in patients with high-risk LACC [24]. Based on the OS and PFS results obtained in that trial, pembrolizumab concomitant to CRT and used as maintenance therapy was approved by the U.S. Food and Drug Administration (FDA) in January 2024 for stage III-IVA disease (FIGO 2014 criteria), and the European Medicines Agency (EMA) also approved pembrolizumab for these same stages. It is worth noting that those patients treated with IMRT/VMAT technique benefited from the addition of pembrolizumab in the forest plot of OS and PFS, with an HR of 0.67 (CI95% 0.47–0.94) and 0.66 (CI95% 0.53–0.82), respectively. Similarly, stratification by total doses > 70 Gy showed improved OS (HR 0.68, CI95% 0.50–0.92) and PFS (0.68, CI95% 0.55–0.85). In comparison to other trials, the KEYNOTE-A18 trials demonstrated the critical role of consistently high-quality BT implementation on clinical outcomes. In that trial, approximately 88% of patients underwent volume-based BT, achieving a median EQD2 D90 HR-CTV of 87 Gy. This dose escalation was achieved through the use of IC/IS applicators in 23% of cases, reflecting a commitment to individualized adaptive treatment approaches in the most modern studies.

However, the rates remained lower compared with those observed in EMBRACE-I, as demonstrated in the control arm, where the 2 y PFS was notably inferior to that of the corresponding EMBRACE I subcohort (57% vs. 71%) (Table 2) [40]. The future direction, as targeted by EMBRACE-II, will emphasize the implementation of advanced BT techniques in approximately 70% of newly diagnosed LACC, particularly within the high-risk LACC population [41]. While the KEYNOTE-A18 trial provides valuable insights into real-world BT practices, it also highlights a significant gap in the consistent application of high-quality BT. At the 2025 ESTRO Congress, the EMBRACE working group presented the overall clinical outcomes from the prospective, multicenter, and interventional EMBRACE II study. In the high-risk subcohort (T3/T4, T1/2 node-positive disease, including para-aortic node involvement), 3- and 5-year OS rates reached 84% and 78%, while PFS rates were 73% and 68%, respectively. These outcomes highlight the critical impact of advanced IGABT with IC/IS components (74% of patients) to achieve disease control [42].

Recently, the results of the academic randomized phase II ATEZOLACC trial were presented at the 2025 ESGO Congress. Despite modern treatment standards, including MRI-IGABT, the addition of atezolizumab did not result in a statistically significant improvement in PFS (70% vs. 64%, HR 0.77 [CI95% 0.46–1.29]). Importantly, the rate of IC/IS BT remained modest (36%), and the median EQD2 HR-CTV D90 dose was slightly below the EMBRACE recommendations (around 84 Gy) (Table 1) [37].

Despite these significant findings, access to IGABT remains very limited in low- and middle-income countries. This has led to the increased use of alternative techniques such as boost with EBRT and stereotactic body radiotherapy (SBRT), which negatively impact survival rates [43]. These findings reinforce the urgent need for global initiatives focused on education, training, and standardization, ensuring that all patients, regardless of geographic location, have access to the highest standards of care. The GYN GEC-ESTRO working group is actively working to provide non-inferior and more cost-effective alternatives to MRI for use in IGABT, such as CT scans and transrectal ultrasounds (TRUS) [44,45,46]. In addition, they are developing fellowship programs to support specialized training in centers of excellence [47].

Another key aspect is the chronic morbidity associated with non-image-guided BT. Historically, severe toxicity rates in patients treated with 2D and 3D BT range from 3.5% to 23%, with a major negative impact on quality of life [48]. The transition from older BT techniques to IGABT has greatly improved the toxicity profile in these patients, with reported 5-year rates (EMBRACE I) of chronic bladder and rectal toxicity ≤ grade (G) 2 ranging from 20 to 25% and ≥G3 toxicity from 3 to 8.5% [12]. In this context, it is important to closely monitor chronic morbidity rates in all these trials, especially given the differences in morbidity between older BT techniques and IGABT. This should provide valuable data to better characterize the impact of suboptimal RT and BT.

In summary, across these major studies, the following consistent pattern emerges: the quality and technique of BT impact survival outcomes and toxicity profiles in LACC. Volume-based IGABT—with the use of IC/IS applicators when needed—is essential.

## 5. Conclusions

The paradigm of LACC management is evolving with the integration of novel systemic therapies; however, the foundation of curative treatment remains focused on high-quality IGABT. Future clinical trials and real-world practice must prioritize the universal implementation of MRI-based volume-adaptive BT, alongside robust radiotherapy quality assurance programs. Only by securing optimal local control through advanced BT techniques can emerging systemic strategies achieve their full potential, ultimately improving survival outcomes and quality of life for patients with LACC.

## Figures and Tables

**Table 1:** Summary of main studies evaluating treatment strategies for LACC comparing the treatment arms and outcomes (overall survival, disease-free survival, and brachytherapy characteristics).

STUDY (Patients)	Treatment Arms	Overall Survival	Disease-Free Survival	Brachytherapy Characteristics	Comments
Pötter et al. 2021 (1381 pt) [4]	RTCHT + MRI-image IGABT	74% (5 y) (78% INTERLACE-LIKE cohort)	68% (5 y) (72% INTERLACE-LIKE cohort)	IGABT CTV-HR (100%) Median dose 90 Gy EQD2 IC/IS 43%	MRI-based IGABT provided high LC with limited morbidity
Mileshkin et al. 2023 Phase III (926) [25]	RTCHT + adjuvant ChT vs. standard RTCHT + BT	72% vs. 71% (5 y)	63% vs. 62% (5 y)	Point A (64%), IGABT (29%), not recorded (7%) Median dose N/A IC/IS not allowed	Adjuvant ChT + RTCHT did not improve OS and PFS Both treatments acceptable
Mc Comarck et al. 2024 Phase III (500) [26]	IChT + RTCHT vs. standard RTCHT + BT	80% vs. 72% (5 y)	PFS: 72% vs. 64% (5 y)	2D (20%), point A (50%), volume-based (30%) Median dose 79.4 Gy EQD2	IChT improved OS and PFS with non-standard BT; no data in late toxicity
Monk et al. 2023 Phase III (770) [27]	RTCHT- Durvalumab + adjuvant Durvalumab vs. standard RTCHT + BT	Not reached	65.9% vs. 62.1% (2 y)	IGABT (62%) Point A (38%) Mean dose 83 Gy EQD2	No significant difference in PFS; the subgroup with N+ and III and/or para-aortic N benefit more
Lorusso et al. 2024 Phase III (1060) [24]	RTCHT- Pembrolizumab + adjuvant Pembrolizumab vs. standard RTCHT + BT	82.6% vs. 74.8% (3 y)	69.3% vs. 56.9% (3 y)	IGABT (88.2%) Point A (9.3%) Mean dose 87 Gy EQD2 IC/IS 23%	Pembrolizumab in concomitance and in maintenance improves statistically PFS and OS manageable safety profile
Chargari et al. abstract 2025 Phase II (189) [29]	RTCHT- Atezolizumab + adjuvant Atezolizumab vs. standard RTCHT + BT	N/A	70% vs. 64% (2 y)	MRI-based IGABT (75%) Median dose D90 HR-CTV 84.4 Gy EQD2 IC/IS 36%	No significant improvement in 2 y PFS in high-risk cervical cancer patients treated with MRI-IGABT and IS/IC

OS—overall survival, BT—brachytherapy, EQD2—equivalent dose in 2 Gy fractions, IGABT—image-guided adaptive brachytherapy, CTV-HR—clinical target volume–high risk, PFS—progression-free survival, 2D—two-dimensional, MRI—magnetic resonance imaging, LC—local control, IChT—induction chemotherapy, RTCHT—concurrent chemoradiation therapy, N—nodal, IC/IS—intracavitary/interstitial.

**Table 2:** Five-year relapse patterns and reported PFS (except in those specified) across phase III studies are presented.

Relapse Patterns	KA-A18 Standard Arm	INTERLACE Standard Arm	INTERLACE Experimental Arm	ATEZOLACC Standard Arm	EMBRACE I Subcohort: IB1 N+, IB2, II, IIIB, IVA	EMBRACE I Subcohort: IB3-IIB N+, III-IVA
Local/pelvic	N/A	17%	17%	11.7%	12%	12%
Distant	N/A	20%	13%	N/A	16%	21% (3 y)
Overall *	N/A	28%	23%	N/A	23%	
PFS	68% (2 y)	64% (5 y)	73% (5 y)	64% (2 y)	72% (5 y)	71% (2 y)

* locoregional and distant. N/A—not available, PFS—progression-free survival, y—year.

## Abbreviations

The following abbreviations are used in this manuscript:

LACC	Locally advanced cervical cancer
IGABT	Image-guided adaptive brachytherapy
RT	Radiation therapy
EMBRACE	IntErnational Study on MRI-Based BRAChytherapy in Cervical Cancer
OS	Overall survival
LC	Local control
ChT	Chemotherapy
PFS	Progression free survival
DFS	Disease free survival
OTT	Overall treatment time
BT	Brachytherapy
RCT	Randomized clinical trial
GEC-ESTRO	Gynecological (GYN) Groupe Europeen De Curietherapie-European Society for Therapeutic Radiology and Oncology
IMRT	Intensity modulated radiation therapy
VMAT	Volumetric arc therapy
MRI	Magnetic resonance imaging
IC	Intracavitary
IC/IS	Intracavitary/interstitial
HDR	High dose rate
PDR	Pulse dose rate
HR-CTV	High risk clinical target volume
IR-CTV	Intermediate risk clinical target volume
EQD2	Equivalent dose in 2 Gy fractions
OAR	Organs at risk
D90	Dose to 90% of the volume
OTT	Overall treatment time
IChT	Induction chemotherapy
LDR	Low dose rate
ICI	Immune checkpoint inhibitors
FDA	Food and drug administration
EMA	European medicines agency
SBRT	Stereotactic body radiation therapy
